# Autolysis of *Pseudomonas aeruginosa* Quorum-Sensing Mutant Is Suppressed by *Staphylococcus aureus* through Iron-Dependent Metabolism

**DOI:** 10.4014/jmb.2312.12028

**Published:** 2024-02-02

**Authors:** Shin-Yae Choi, In-Young Chung, Hee-Won Bae, You-Hee Cho

**Affiliations:** Program of Biopharmaceutical Science and Department of Pharmacy, College of Pharmacy and Institute of Pharmaceutical Sciences, CHA University, Gyeonggi-do 13488, Republic of Korea

**Keywords:** *Pseudomonas aeruginosa*, *Staphylococcus aureus*, quorum-sensing, iron, secretome

## Abstract

Microorganisms usually coexist as a multifaceted polymicrobial community in the natural habitats and at mucosal sites of the human body. Two opportunistic human pathogens, *Pseudomonas aeruginosa* and *Staphylococcus aureus* commonly coexist in the bacterial infections for hospitalized and/or immunocompromised patients. Here, we observed that autolysis of the *P. aeruginosa* quorum-sensing (QS) mutant (*lasRmvfR*) was suppressed by the presence of the *S. aureus* cells in vitro. The QS mutant still displayed killing against *S. aureus* cells, suggesting the link between the *S. aureus*-killing activity and the autolysis suppression. Independent screens of the *P. aeruginosa* transposon mutants defective in the *S. aureus*-killing and the *S. aureus* transposon mutants devoid of the autolysis suppression revealed the genetic link between both phenotypes, suggesting that the iron-dependent metabolism involving *S. aureus* exoproteins might be central to both phenotypes. The autolysis was suppressed by iron treatment as well. These results suggest that the interaction between *P. aeruginosa* and *S. aureus* might be governed by mechanisms that necessitate the QS circuitry as well as the metabolism involving the extracellular iron resources during the polymicrobial infections in the human airway.

## Introduction

Microorganisms usually coexist with a wide variety of polymicrobial communities, not only on abiotic surfaces in their natural habitat, but also on mucosal sites in the human body. These polymicrobial populations from resident microbiota and/or invading microbes can be regarded as important determinants of the human health and physiology, whose imbalances may lead to the pathological states of the human bodies. In recent years, increased attention has been paid to the complex interactions that occur in the polymicrobial populations, especially during bacterial infections. One of the best studied polymicrobial infections caused by complex communities of bacterial pathogens is the respiratory infections in the patients with cystic fibrosis (CF), where four major bacterial species (*i.e.*, *Pseudomonas aeruginosa*, *Staphylococcus aureus*, *Haemophilus influenzae*, and *Burkholderia cepacia* complex) have been focused on [1, 2], with the two most prevalent species being *P. aeruginosa* and *S. aureus* [3]. They are highly notorious ESKAPE pathogens and the well-studied model bacteria in various microbiological aspects [4]. *P. aeruginosa* is a Gram-negative bacterium that is commonly found in soil and water as well as in plants, animals, and humans. *P. aeruginosa* has become an emerging opportunistic pathogen with multiple antibiotic resistance and tolerance in the clinics. *S. aureus* is a Gram-positive bacterium that is identified from warm-blooded animals, being a common cause of food poisoning and skin infections such as abscesses. Methicillin-resistant *S. aureus* (MRSA) is a worldwide concern in clinical medicine.

It is recently known that both *P. aeruginosa* and *S. aureus* affect each other in mixed cultures in vitro [5, 6]. *P. aeruginosa* produces 2-heptyl-4-hydroxyquinoline-*N*-oxide (HQNO) that kills *S. aureus* cells most likely to survive physiological conditions depleted in irons [7, 8]. HQNO-mediated *S. aureus*-killing is attributed to the apparent disturbance of electron transport in *S. aureus*. Given that functional electron transport chains are required not only to support the rapid growth of *S. aureus*, but also to render *S. aureus* cells susceptible to the bactericidal antibiotics [9], HQNO could select *S. aureus* small colony variants with altered respiratory activity in the presence of antibiotics during the interaction with *P. aeruginosa* [10]. They suggest the complex interactions between these two bacterial species during human infections and antibiotic treatment.

In this study, we first observed that the *P. aeruginosa* quorum-sensing (QS) mutant (*lasRmvfR*) devoid of HQNO production still displayed some residual killing activity against *S. aureus* mutants with altered respiratory activity. The QS mutant suffers from earlier autolysis than the wild type, which was suppressed by the presence of *S. aureus* cells as well as their culture supernatants. We have also uncovered the genetic link between the *S. aureus*-killing activity of *P. aeruginosa* and the autolysis suppression by *S. aureus*, based on the identification of the genes from both bacterial species, which might contribute to iron-dependent metabolism in *P. aeruginosa* and exoprotein secretion in *S. aureus*.

## Materials and Methods

### Bacterial Strains and Culture Conditions

The bacterial strains and plasmids used in this study are described in Table 1. Luria-Bertani (LB) (1% tryptone, 0.5% yeast extract and 1% NaCl) broth, LB broth supplemented with 50 mM KNO_3_ (LBN), Tryptic soy broth (Difco, USA), 2% Bacto-agar (Difco) LB plates, and cetrimide agar (CA) (Difco) plates were used. Overnight-grown cultures were used as inoculum (1% sub-culture) into fresh broth and grown at 37°C in a shaking incubator until the logarithmic growth phase (*i.e.*, OD_600_ of 1.0), and then the cell cultures were used for the experiments described herein.

### Construction of Deletion Mutants

All the deletion constructs were created using pEX18T as described elsewhere [16]. Oligonucleotide primers were designed using the PA14 genome sequence. SOEing (splicing by overlap extension) PCR was conducted by using four oligonucleotide primers for in-frame deletions as listed in Table 2. The resulting constructs were introduced into the wild type PA14 or the relevant mutants like the QS (*lasRmvfR*) mutant and the double-crossover recombinants were obtained by sucrose selection from the cointegrates, all of which were verified by PCR at each stage.

### Autolysis Assay

*P. aeruginosa* autolysis was examined in 24-h or 48-h LBN cultures. Briefly, freshly grown (OD_600_ of 1.0) cells (~10^6^ CFU) of *P. aeruginosa* or *S. aureus* were inoculated into the 48-well plate wells containing 400 μl LBN broth. The plates are incubated on a rotatory shaker at 37°C for either 24 or 48 h. Autolysis is monitored by visual inspection of aggregated cell debris, which was verified by Live/Dead-Baclight staining (Invitrogen, USA) [33].

### *S. aureus* Killing Assay

*S. aureus* killing was assessed either by plate killing or by growth competition in 16-h liquid culture. Plate killing assay was previously described [18]. Briefly, LBN plates were overlaid with 0.7% top agar containing 100 μl of *S. aureus* cultures that had been grown to OD_600_ of 1.0 and then dried for 1 h under sterile air blowing. *P. aeruginosa* bacterial suspensions (3 μl) containing 10^6^ CFU of early stationary growth phase (OD_600_ of 3.0) were spotted onto the *S. aureus* lawns. Plates were incubated at 37°C for 16 h. The killing activity is scored as the visible halo around the cell spots.

Growth competition was monitored by separate viable counts of each strain after 16-h coculture of *P. aeruginosa* and *S. aureus*. Freshly grown (OD_600_ of 1.0) cells (10^6^ CFU) of *P. aeruginosa* and *S. aureus* were inoculated into the culture tubes containing 3 ml LBN broth. After 16-h incubation, culture suspensions were 10-fold serially diluted, and the diluted samples (3 μl) were spotted onto the LB agar plates containing either 5% NaCl (for *S. aureus* selection) and 50 μg/ml rifampicin (for *P. aeruginosa* selection).

### Transposon Experiments

Two plasmids (pBTK30 with *Himar1* for *P. aeruginosa* and pTV1 with Tn*917* for *S. aureus*) were used for transposon mutagenesis [34, 35]. pBTK30 was introduced into the *P. aeruginosa* QS mutant by conjugation for 5 h at 37°C. CA plates containing gentamicin (50 μg/ml) were used for *Himar1* transposant selection. A total of 1,734 transposon insertion clones were screened for the mutants devoid of the residual *S. aureus*-killing activity of the QS mutant. Three mutants were chosen out of the 67 primary candidates. pTV1 was introduced into *S. aureus* *m5* by transformation. Temperature induction and selection was performed by growing the cells overnight in LB broth supplemented with 10 μg/ml erythromycin at 42°C. A total of 1,765 chloramphenicol-sensitive and erythromycin-resistant Tn*917* transposant clones were screened for the mutants incapable of the autolysis suppression, resulting in 2 transposon clones as the final candidates. The transposon insertion sites were determined by arbitrary PCR followed by sequencing using the appropriate primers listed in Table 2.

### Protein Experiments

Exoprotein profiles were analyzed by SDS-PAGE. *S. aureus* cells were grown, and the culture supernatants were precipitated by 10% (v/v) trichloroacetic acid and separated on a 12% (vol/vol) polyacrylamide gel at 100 V for 110 min. The gels were stained with Coomassie Brilliant blue R 250 for 30 min as described elsewhere [36]. Ammonium sulfate (AS) precipitation was used for partial fractionation of the exoproteins, by altering the AS concentrations. The culture supernatant from 500 ml of the *S. aureus* *m5* culture was subjected to filtration (0.22-μm membrane filter) and the filtrate was mixed with cOmplete^TM^ ULTRA Tablets EASYpack (Roche, Switzerland) and then kept at 4°C for 16 h. The sample was transferred to a beaker and AS powder was gradually added while agitating the sample to reach a final concentration of 50%. The sample was subjected to centrifugation at 12 K for 30 min to separate the pellet and the supernatant. The pellet was dissolved in PBS buffer (2.7 mM KCl, 137 mM NaCl, 10 mM Na_2_HPO_4_, and 2 mM KH_2_PO_4_, pH 7.0) as the 50% AS sample. The supernatant was further subjected to the AS addition to reach 65% and the 65% AS sample was obtained after dissolution of the 65% AS pellet. Likewise, the 80% and the 90% AS samples were obtained.

## Results and Discussion

### *P. aeruginosa* Autolysis Is Suppressed by *S. aureus*

**Fig. 1:**
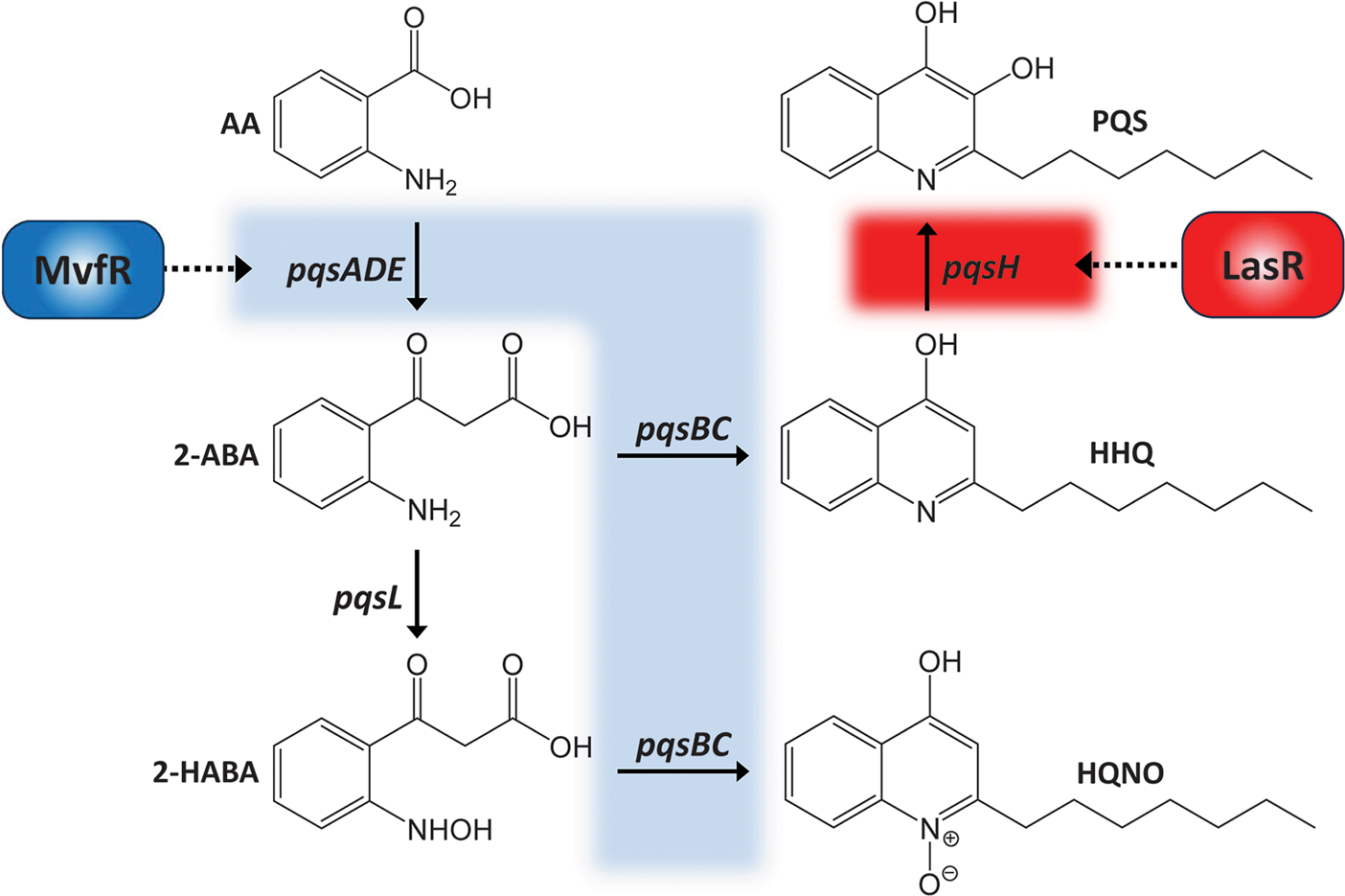
Schematic overview of the PQS biosynthesis. The biosynthetic enzymes for the PQS-related signaling molecules are encoded by the *pqs* genes as indicated, and their expression is positively regulated by the QS-regulators LasR and MvfR as highlighted in red and blue, respectively. Abbreviations for the molecules: AA, anthranilic acid; 2-ABA, 2'-aminobenzoylacetic acid; 2-HABA, 2'-hydroxylaminobenzoylacetatic acid; HHQ, 4-hydroxy-2-heptylquinoline; HQNO, 2-heptyl-4-hydroxyquinoline- *N*-oxide; PQS, *Pseudomonas* quinolone signal.

Autolysis phenotype of *P. aeruginosa* has long been known, which are associated with the overproduction of the PQS-related signaling molecules [11]. This phenotype is frequently observed in the clinical isolates from the CF patients, which is attributed to the mutations in the *lasR* gene encoding the master QS regulator in *P. aeruginosa*. Fig. 1 represents the involvement of the QS regulators, LasR and MvfR (PqsR), in regulation of the biosynthetic pathway for the PQS-related signaling molecules: the *pqsABCD* and *pqsE* genes are positively regulated by MvfR, whereas the *pqsH* gene is under the LasR control. Autolysis is often observed in the prolonged incubation of the *lasR* mutant of the laboratory strains [12], which undergoes accumulation of the PQS precursor, 2-heptyl-4-hydroxyquinoline (HHQ) and/or HQNO [13]. It is known that the wild-type *P. aeruginosa* laboratory strains (PA14 and PAO1) exhibits autolysis after 48-h growth in the planktonic cultures, which is triggered by HQNO-mediated self-poisoning of the electron transport chains [14]. HQNO also poisons *S. aureus* at the level that could not poison *P. aeruginosa*. It can select for the small colony variants (SCVs) of *S. aureus* in condition that the growth of both *P. aeruginosa* and *S. aureus* could be affected by antibiotics in the mixed culture [10, 15].

**Fig. 2:**
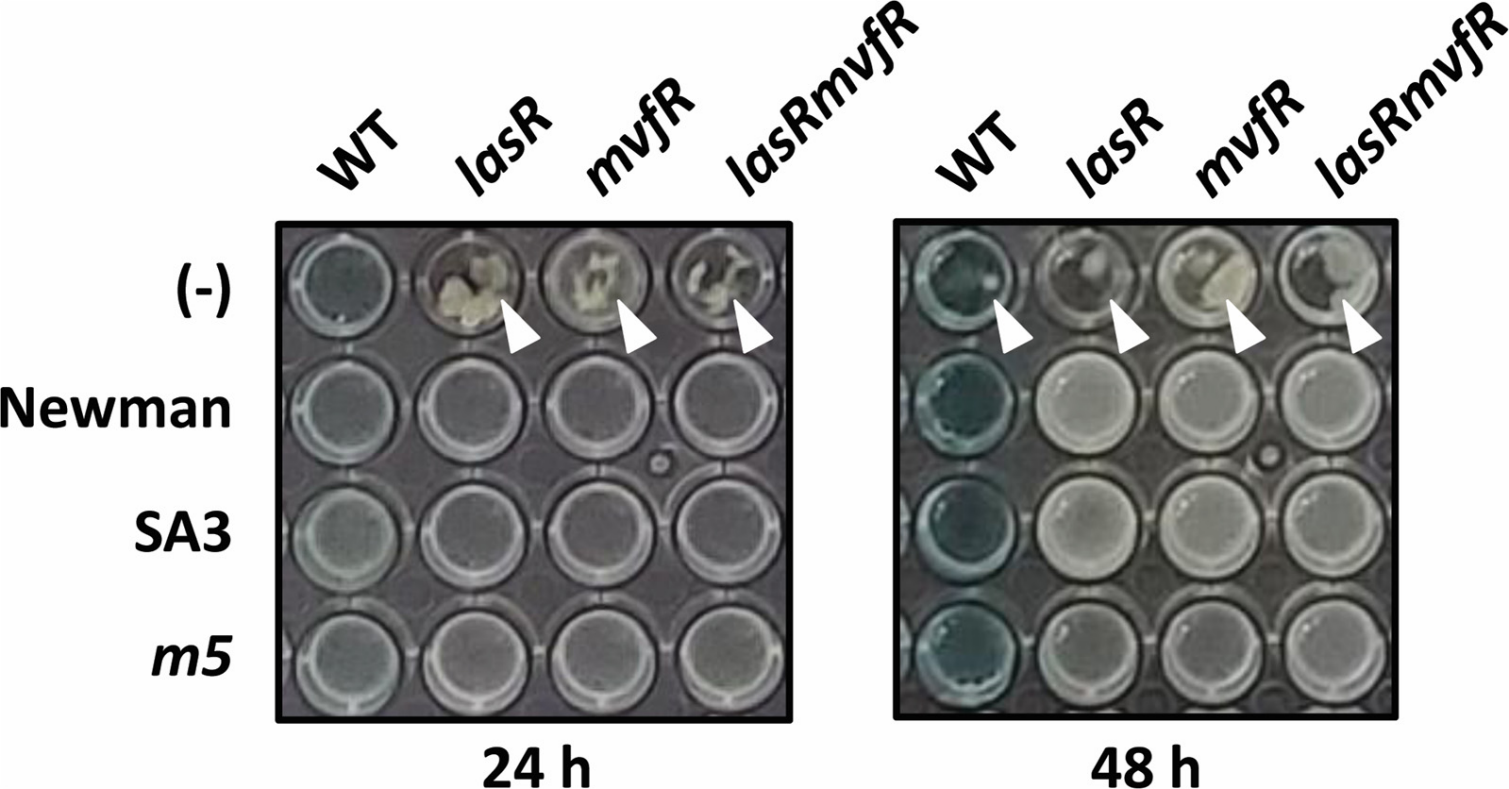
Autolysis suppression of the QS mutants. Autolysis of *P. aeruginosa* PA14 (WT) and its QS mutants (*lasR*, *mvfR*, and *lasRmvfR*) was monitored from the 48-well LBN cultures in the presence or absence (-) of *S. aureus* strains (Newman, SA3, and *m5*), which were grown at 37°C for either 24 h or 48 h. Cell debris by autolysis is indicated by arrowheads.

To better understand the autolysis phenotype of *P. aeruginosa* regarding QS-dependent and/or conditional HQNO poisoning of both species, we used the QS mutants of *P. aeruginosa* PA14 during the coculture with *S. aureus* strains under aerobic nitrate-respiration condition, which might promote alternative respiration mode [16]. Fig. 2 shows that the wild type (WT) underwent visible autolysis with pigment overproduction by 48-h incubation, but not by 24-h incubation, whereas the QS mutants such as *lasR*, *mvfR*, and *lasRmvfR* displayed autolysis phenotypes by 24-h incubation. This result suggests that the *P. aeruginosa* QS involving LasR and MvfR is required to delay the autolysis, although they are required to generate the known auto-poisoning molecule, HQNO. This observation of the time-dependent relationship between autolysis and HQNO would be attributed to the differential susceptibility of the QS mutants to HQNO and/or to the other unknown mechanisms by which the earlier autolysis should occur in the QS mutants.

Michelsen *et al*. [15] reported the commensal-like interaction between *P. aeruginosa* and *S. aureus*, in which *S. aureus* was not killed by a certain *P. aeruginosa* isolate and its autolysis was suppressed by *S. aureus*. This study prompted us to evaluate the QS mutants for the *S. aureus*-mediated autolysis suppression, in that the HQNO-directed *S. aureus*-killing activity should be clearly reduced in the QS mutants. The WT *S. aureus*, Newman, an MRSA strain, SA3, and its respiratory mutant (*m5*) were used for the coculture with *P. aeruginosa*. The growth of *m5* is comparable to that of SA3, and the whole genome sequencing revealed that *m5* contains the *ubiE* mutation for the ubiquinone metabolism and two other mutations (*atl_2* and *lytN_1*) [17]. We revealed that all the *S. aureus* strains were able to suppress the autolysis of the QS mutants and the 48-h culture autolysis of the WT. This and the fact that the WT *P. aeruginosa* can kill *S. aureus*, not in such commensal-like interactions, led us to hypothesize that the QS mutants could still kill *S. aureus*, which might be associated with the autolysis suppression of *S. aureus*.

### *P. aeruginosa* Autolysis Suppression Is associated with the Residual *S. aureus*-Killing Activity

**Fig. 3:**
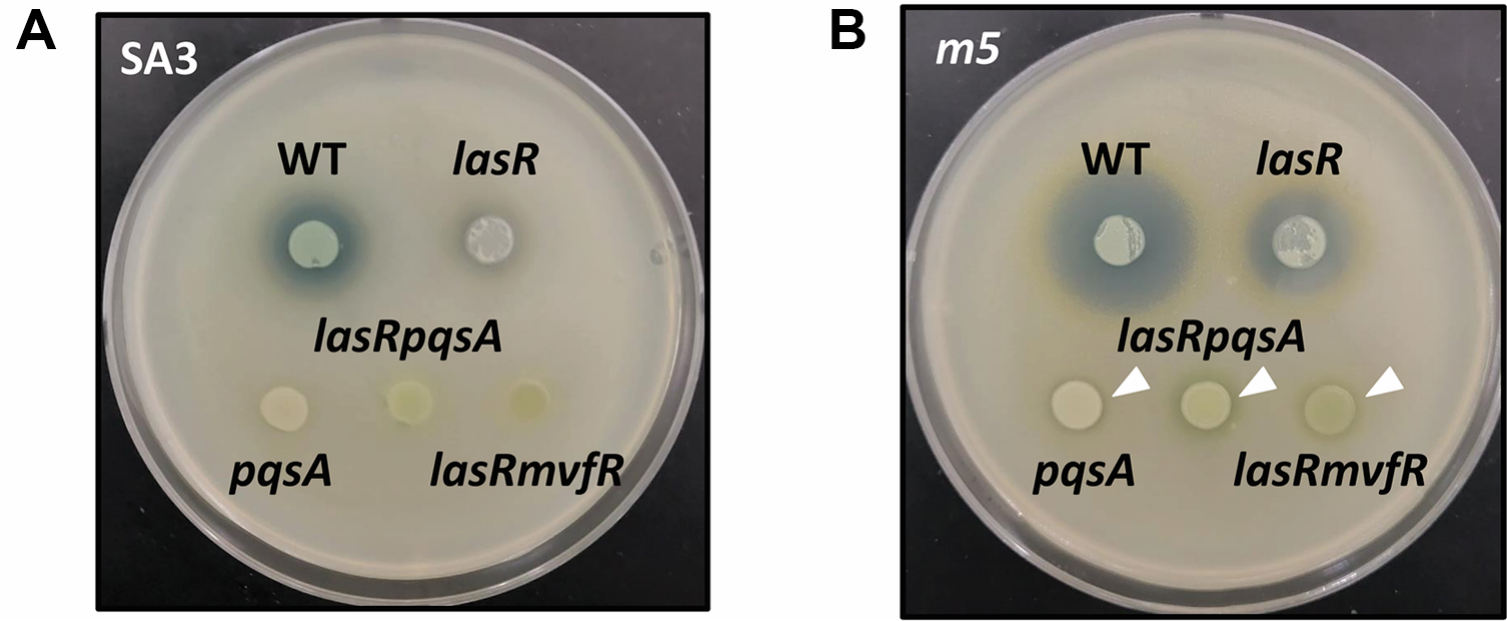
*S. aureus* killing of the QS mutants. *S. aureus* killing of *P. aeruginosa* PA14 (WT) and its mutants (*pqsA*, *lasRpqsA*, and *lasRmvfR*) was monitored on the cell lawns of *S. aureus* SA3 (**A**) and *m5* (**B**). The residual killing activity is indicated by arrowheads.

*P. aeruginosa* *mvfR* and *pqsA* mutants are impaired in killing Gram-positive bacteria [18]. It is noted that some residual killing activity happened to be observed on the respiratory mutant, *m5* (Fig. 3). The killing activity was simply assessed by spotting *P. aeruginosa* cells on the lawns of Gram-positive bacteria. *m5* is one of the five mutants that are resistant to naphthoquinone-generated reactive oxygen species (ROS) and have a *ubiE* mutation in common [17]. Those five mutants showed reduced respiratory activity and subsequently reduced ROS generation [17], where ROS would be the key to HQNO-poisoning of *S. aureus*. Fig. 3A shows that the *S. aureus*-killing activity was observed by the WT *P. aeruginosa* and, to the lesser extent, by the *lasR* mutant. However, the mutations in *mvfR* and *pqsA* completely abolished the visible killing activity on SA3. This is consistent with the previous observation that HQNO is crucial to the *S. aureus*-killing activity [10]. However, we highlighted some residual killing activity on the *m5* mutant for the killing assay (Fig. 3B). The killing by the WT as well as that by the *lasR* mutant were also enhanced on *m5*, suggesting HQNO is the major, but not the sole killing activity against *S. aureus*. The residual killing activity was scarcely detected previously, but evident when *P. aeruginosa* interacts with the *m5* mutant of *S. aureus*. The residual killing activity must have been unseen in the *S. aureus* with normal respiration. Although it needs to be further verified that the altered respiration as observed in *m5* could occur during the natural interaction between *P. aeruginosa* and *S. aureus*, we hypothesized that the *P. aeruginosa* autolysis suppression by *S. aureus* might be associated with the residual *S. aureus*-killing activity by *P. aeruginosa*.

### *P. aeruginosa* Autolysis Suppression and *S. aureus*-Killing Activity Are Genetically Linked

**Fig. 4:**
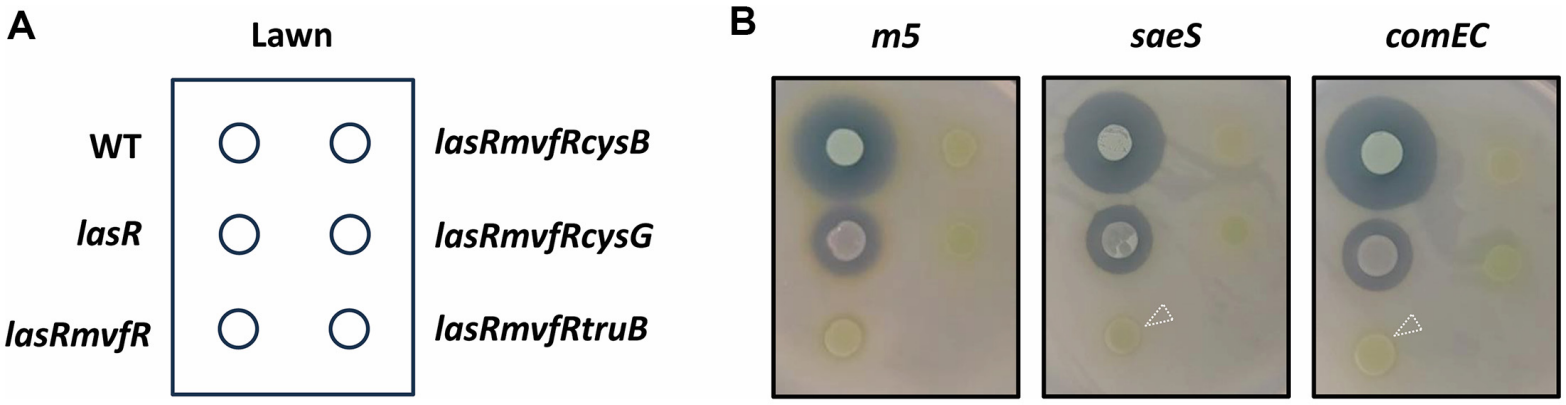
*S. aureus* killing of the isolated mutants. (**A**) Positions of *P. aeruginosa* PA14 (WT) and its mutant (*lasR*, *lasRmvfR*, *lasRmvfRcysB*, *lasRmvfRcysG*, and *lasRmvfRtruB*) cells spots in **B** that had been applied to *S. aureus* killing plate assay as in Fig. 3. (**B**) *S. aureus* killing of *P. aeruginosa* cells as in **A** was monitored on the cell lawns of *S. aureus* *m5* and its mutants (*saeS* and *comEC*). The disappearance of the residual killing activity of *lasRmvfR* is indicated by dotted arrowheads.

To validate the association between the autolysis suppression and the residual *S. aureus*-killing activity, we attempted to isolate the mutants from both *P. aeruginosa* and *S. aureus*, which are defective in the residual *S. aureus*-killing activity and the autolysis suppression, respectively. Random transposon mutagenesis as described in Materials and Methods enabled us to identify three mutants of *P. aeruginosa* *lasRmvfR* (*cysB*, *cysG*, and *truB*) and two mutants of *S. aureus* *m5* (*saeS* and *comEC*) (Fig. 4). It is important at this stage that we just wanted to gain an insight into the relationship between the autolysis suppression and the residual *S. aureus*-killing activity. Therefore, we did not delve into characterization of the individual genes, but just wanted to validate the genetic link therebetween.

The involvement of the *truB* gene that encodes a subunit of pseudouridine synthase in tRNA modification [19] might be surprising. It should be noted, however, that the *truA* gene encoding another subunit of pseudouridine synthase is required for the optimal expression of type III secretory genes presumably by affecting the tRNA-mediated translation efficiency [20], suggesting that some TruB-dependent tRNA functions might be required for the optimal expression of the genes involved in the residual killing activity in *P. aeruginosa*.

The *cysB* gene encodes the master regulator (CysB) in sulfur uptake and cysteine biosynthesis [21], whereas the *cysG* encodes a methyltransferase for siroheme biosynthesis [22], despite its sequence and functional similarity to methyltransferases, NirE and CobA, in heme *d1* and cobalamin synthesis, respectively [23]. Although we have not performed complementation experiments and do not fully understand whether these genes are indeed involved in the residual *S. aureus*-killing activity, the involvement of CysG was noteworthy in that it is associated with nitrate respiration and sulfur metabolism, in that siroheme is the prosthetic group for both nitrite reductase and sulfite reductase [24]. The impact of siroheme in addition to the previous finding that *P. aeruginosa* uses *S. aureus* as the iron source upon *S. aureus* killing [5] suggest the importance of iron-dependent metabolism of *P. aeruginosa* in the residual *S. aureus*-killing activity.

Identification of the *S. aureus* *m5* mutants (*saeS* and *comEC*) also revealed the importance of virulence exoproteins (for *saeS*) and membrane functions (for *comEC*) in support for the *S. aureus*-killing. SaeS is a histidine kinase of the two-component regulatory system (SaeRS) that is required for expression and secretion of various extracellular virulence factors [25], whereas *comEC* encodes a large integral membrane protein forming a large channel for passage of DNA and/or peptides for competence [26]. Although we did not experimentally verify either whether these are indeed required for the residual *S. aureus*-killing activity, it is evident that the isolated mutant clones were devoid of the residual *S. aureus*-killing activity (Fig. 4).

**Fig. 5:**
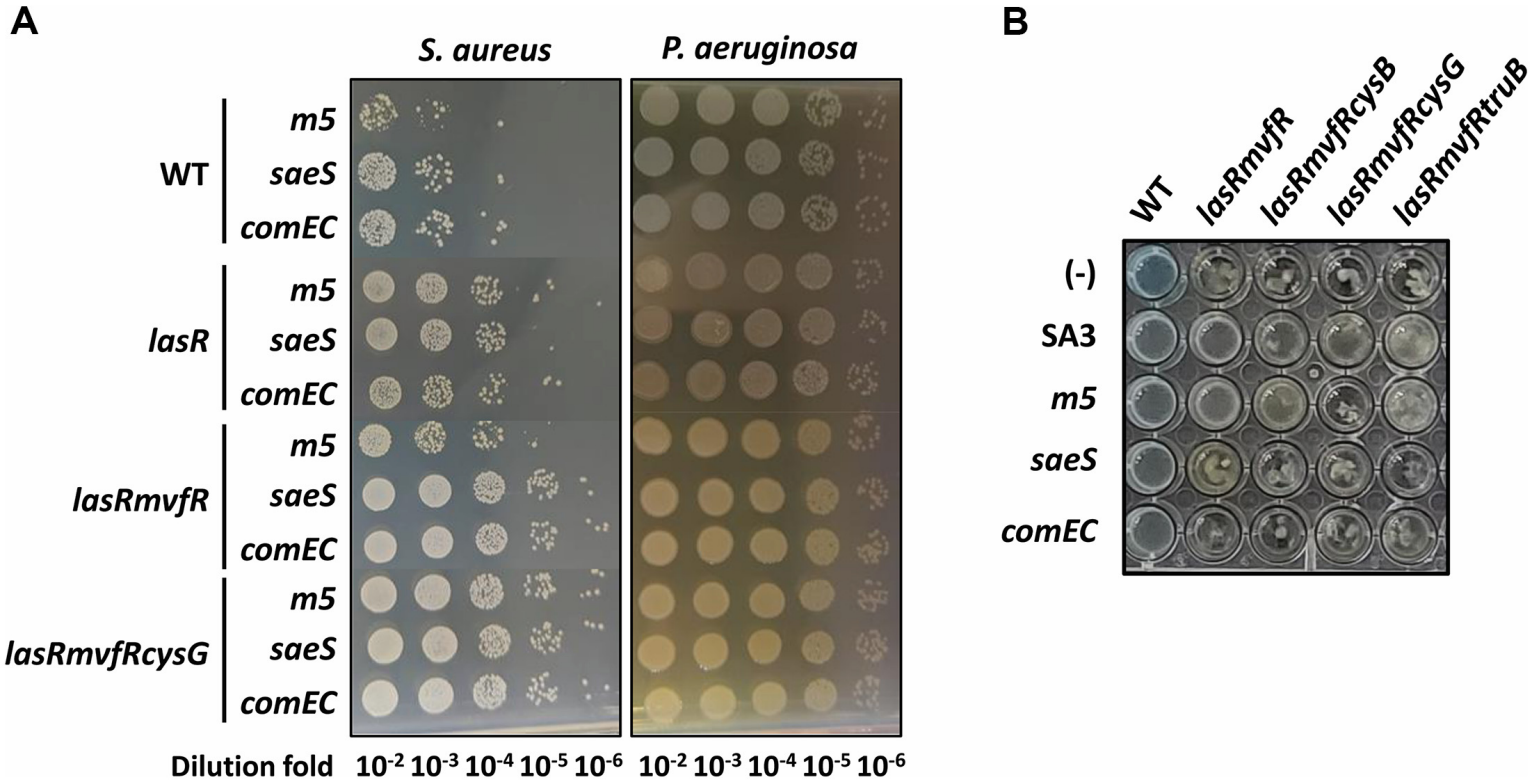
*S. aureus*-killing and autolysis suppression of the isolated mutants. (**A**) *S. aureus* killing of *P. aeruginosa* PA14 (WT) and its mutants (*lasR*, *lasRmvfR*, and *lasRmvfRcysG*) cells was monitored after growth competition between one of them and *S. aureus* (*m5*, *saeS*, and *comEC*) in 24-h liquid culture. Culture suspensions were diluted and spotted on LB plates amended with either 5% NaCl (to select *S. aureus*) or 50 μg/ml rifampicin (to select *P. aeruginosa*). The numbers indicate the dilution folds of the culture suspension. (**B**) Autolysis of *P. aeruginosa* PA14 (WT) and its mutants (*lasRmvfR*, *lasRmvfRcysB*, *lasRmvfRcysG*, and *lasRmvfRtruB*) was monitored from the 48-well LBN cultures in the presence or absence (-) of *S. aureus* strains (SA3, *m5*, *saeS* and *comEC*), which were grown at 37°C for 24 h.

To verify the *S. aureus* killing quantitatively, we designed the mixed culture of these two species in liquid broth. After 16-h culture, the remaining bacteria were enumerated by separate viable counts on the selective media as described in Materials and Methods. Fig. 5A shows the viable counts of *S. aureus* and *P. aeruginosa* after the coculture: in all cultures, the growth of *P. aeruginosa* was not affected at all by the presence of *S. aureus*. As expected, however, the killing activity against *m5* was highest in the WT and no killing activity was observed in the *lasRmvfRcysG* mutant. In contrast, only the residual killing activity of the *lasRmvfR* mutant disappeared in either *P. aeruginosa* mutation (*cysG*) or *S. aureus* mutations (*saeS* and *comEC*), suggesting that both CysG and SaeS (or ComEC) are simultaneously required for the *m5*-killing activity of the *lasRmvfR* mutant.

To verify the genetic link between the autolysis suppression and the residual *S. aureus*-killing activity, these mutants were tested for their ability to suppress the autolysis (Fig. 5B). It is noted that *saeS* and *comEC* bacteria could not suppress the autolysis of the *m5*-killing QS mutant and that the *m5* bacteria could not suppress the autolysis of the unkilling QS mutants (especially with *cysG*). These results substantiate the genetic link between the autolysis suppression and the residual *S. aureus*-killing activity.

### *P. aeruginosa* Autolysis Is Suppressed by *S. aureus* Exoproteins or Iron Treatment

**Fig. 6:**
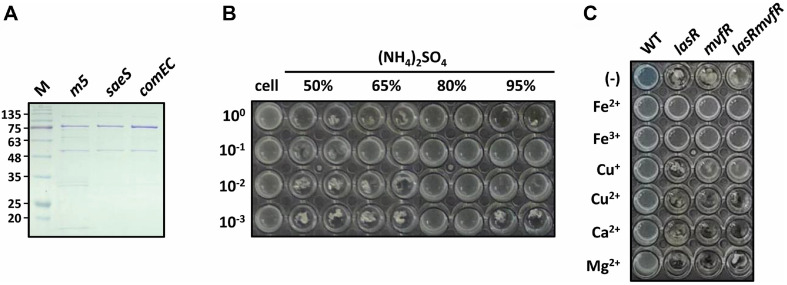
Autolysis suppression of the extracellular proteins and metals. (**A**) Profiles of extracellular proteins from *S. aureus* *m5* and its mutants (*saeS* and *comEC*) were analyzed by 12% SDS-PAGE. The size markers (**M**) are included with the molecular weight (kDa) of representative bands. (**B** and **C**) Autolysis of *P. aeruginosa* PA14 was monitored from the 24-h 48- well LBN cultures in the presence of either ammonium sulfate ((NH_4_)_2_SO_4_) precipitate fractions (**B**) at the indicated concentrations (50%, 65%, 80%, and 95%) or metal ions (**C**).

Based on the genetic link between autolysis suppression and the residual *S. aureus*-killing activity featuring the identified genes especially the *P. aeruginosa* *cysG* and the *S. aureus* *saeS*, we postulated that the iron metabolism of *P. aeruginosa* and the secreted exoproteins of *S. aureus* could be involved in those phenotypes. It is evident that the extracellular protein profiles differed between the *m5* and the mutant bacteria (Fig. 6A), in that some bands in the *m5* sample were missing in those of the mutants, suggesting that some extracellular proteins could suppress the autolysis of the QS mutant and the subsequent *S. aureus* killing. To confirm this, we obtained the culture supernatant from *m5* and prepared its ammonium sulfate (AS) fractions, which were tested for their ability to suppress the autolysis. As shown in Fig. 6B, the most prominent autolysis suppression was observed in the 80% AS fraction, suggesting that the suppressing activity was enriched in this fraction. The red color of the fraction led us to hypothesize that some iron-containing protein(s) could be the key to the autolysis suppression. Under consideration of the aforementioned mutant screens from *P. aeruginosa* and *S. aureus* in addition to the assumption that the exoprotein(s) would be important in the autolysis suppression, we examined if the treatment with only iron could suppress the autolysis phenotype (Fig. 6C). As a result, both iron (II) and iron (III) could suppress the autolysis of the QS mutants, whereas copper (II), calcium, and magnesium could not. It should be noted that copper (I) could partially suppress the autolysis of the *mvfR* and the *lasRmvfR* mutants, which could be further verified in comparison with other monovalent cations.

Iron is an essential element for growth and survival of most microorganisms. It is involved in many cellular processes as a cofactor tightly coordinated by hemes or amino acid residues of iron-containing proteins. Mashburn *et al*. [7] suggested that *P. aeruginosa* can utilize the iron-containing proteins of *S. aureus* as an iron source, which could be released from the lysed *S. aureus* cells. However, the *S. aureus*-killing activity could vary in the contexts of the interactions between these two species as well as their interactions with the human host in vivo. The involvement of the secreted exoproteins of *S. aureus* in the residual self-killing activity through iron-dependent metabolisms in *P. aeruginosa* needs to be further elucidated by characterizing the chemical identity of both the residual *S. aureus*-killing substance(s) of *P. aeruginosa* and the secreted exoprotein(s) of *S. aureus* that is enriched in the 80% AS fraction.

## Conclusion

Polymicrobial infections can have profound effects on the course, severity, and treatment of microbial infections [27]. In many cases, different microorganisms within a polymicrobial community can lead to facilitated host colonization, enhanced pathogenic potential, and differential immune response [28, 29]. One of the well-studied examples is the interaction between *P. aeruginosa* and *S. aureus* that are highly prevalent in the CF lung and chronic wound infections [3, 30]. In the present study, we demonstrated the apparent association between the residual (not the major) *S. aureus*-killing activity of *P. aeruginosa* and the *P. aeruginosa* autolysis-suppression by *S. aureus*. We first elucidated the residual *S. aureus*-killing activity of the *P. aeruginosa* QS mutant (*lasRmvfR*) by exploiting the respiratory mutant of *S. aureus*, which had been resistant to chemical-generated ROS under aerobic conditions [17].

The connection between electron transport chains and ROS susceptibility is understandable, given that ROS can be generated during the respiration processes on molecular oxygen. It is noted that HQNO, whose production and secretion are controlled by the *P. aeruginosa* QS system, is implicated in both the *S. aureus*-killing and the *P. aeruginosa* autolysis. The finding of the residual *S. aureus*-killing activity in the absence of HQNO enabled us to highlight the importance of iron metabolism during the interaction between *P. aeruginosa* and *S. aureus*, which require the iron-related (*i.e.*, siroheme) metabolism of *P. aeruginosa* and presumably the iron-containing exoprotein(s) of *S. aureus*. It is well known that *P. aeruginosa* can use *S. aureus* as an iron source [7]. The recent identification of pyochelin biotransformation by a secreted enzyme of *S. aureus* [31] also substantiates the importance of iron-dependent metabolism in the *P. aeruginosa*-*S. aureus* interaction. It is still likely that the details of iron availability will vary depending on the structure of the polymicrobial community of *P. aeruginosa* and *S. aureus*, which are more complicated by generation of the SCVs of both species [32]. Nevertheless, this study suggests that the interaction between *P. aeruginosa* and *S. aureus* might be governed by sophisticated mechanisms that necessitate the *P. aeruginosa* QS circuitry and the polymicrobial metabolism involving the extracellular iron resources during the coexistence with *S. aureus* in human airways.

## Figures and Tables

**Table 1:** Bacterial strains and plasmids used in this study.

Strain or plasmid	Relevant characteristics or purpose^a^	Reference or source
*Pseudomonas aeruginosa*		
PA14	Wild type laboratory strain; Rif^R^	Laboratory collection
PA14 *lasR*	PA14 with in-frame deletion of *lasR*; Rif^R^	[18]
PA14 *mvfR*	PA14 with in-frame deletion of *mvfR*; Rif^R^	This study
PA14 *pqsA*	PA14 with in-frame deletion of *pqsA*; Rif^R^	[37]
PA14 *lasRpqsA*	PA14 with in-frame deletion of *lasRpqsA*; Rif^R^	This study
PA14 *lasRmvfR*	PA14 with in-frame deletion of *lasRmvfR*; Rif^R^	This study
PA14 *lasRmvfRcysB*	PA14 with in-frame deletion of *lasRmvfRcysB*; Rif^R^	This study
PA14 *lasRmvfRcysG*	PA14 with in-frame deletion of *lasRmvfRcysG*; Rif^R^	This study
PA14 *lasRmvfRtruB*	PA14 with in-frame deletion of *lasRmvfRtruB*; Rif^R^	This study
*Staphylococcus aureus*		
Newman	Wild type laboratory strain (MSSA); methicillin-sensitive	Laboratory collection
SA3	Wild type laboratory strain (MRSA); Mc^R^	Laboratory collection
*m5*	Respiratory mutant of SA3; Mc^R^	[17]
*m5* *saeS*	*m5* with Tn*917* insertion in *saeS*; Mc^R^ Em^R^	This study
*m5* *comEC*	*m5* with Tn*917* insertion in *comEC*; Mc^R^ Em^R^	This study
*Escherichia coli*		
DH5α	Multipurpose cloning	Laboratory collection
S17-1(λ*pir*)	Conjugal transfer of mobilizable plasmids	Laboratory collection
S17-1(λ*pir*)(pBTK30)	S17-1(λ*pir*) harboring pBTK30 for mariner transposon insertion; Gm^R^ Cb^R^	Laboratory collection
Plasmids		
pEX18T	Allelic exchange by homologous recombination	Laboratory collection
pTV1	Insertional mutagenesis by the transposon Tn*917*; Cm^R^ Em^R^	[35]
pEX18T-cysB	pEX18T with in-frame deletion in the *cysB* gene; Cb^R^	This study
pEX18T-cysG	pEX18T with in-frame deletion in the *cysG* gene; Cb^R^	This study
pEX18T-truB	pEX18T with in-frame deletion in the *truB* gene; Cb^R^	This study

^a^Rif^R^, rifampicin-resistant; Mc^R^, methicillin-resistant; Gm^R^, gentamicin-resistant; Cb^R^, carbenicillin- and ampicillin-resistant; Cm^R^, chloramphenicol-resistant; Em^R^, erythromycin-resistant

**Table 2:** Primers and probes used in this study.

Primer or probe	Relevant characteristics or purpose	Oligonucleotide sequence (5'–3')^a^
*mvfR*-OF	SOEing PCR for *mvfR* in-frame deletion	GAATTCACGAGCAATATGA
*mvfR*-IR	SOEing PCR for *mvfR* in-frame deletion	CGCGCAGGCGCTGGGCGATGACCTGGAGGAA
*mvfR*-IF	SOEing PCR for *mvfR* in-frame deletion	CCAGGTCATCGCCCAGCGCCTGCGCGAACTGG
*mvfR*-OR	SOEing PCR for *mvfR* in-frame deletion	CTGCAGCATGGCAAGAGC
*cysB*-OF	SOEing PCR for *cysB* in-frame deletion	GAATTCGCAGGCTGGATGGTC
*cysB*-IR	SOEing PCR for *cysB* in-frame deletion	GGAGGACGAACTGGGCGGCTTCATGTGCGACT
*cysB*-IF	SOEing PCR for *cysB* in-frame deletion	AGTCGCACATGAAGCCGCCCAGTTCGTCCTCC
*cysB*-OR	SOEing PCR for *cysB* in-frame deletion	GGATCCTCGCCGGCAGCCATA
*cysG*-OF	SOEing PCR for *cysG* in-frame deletion	GGTACCCAGCCAGGACAAGTAC
*cysG*-IR	SOEing PCR for *cysG* in-frame deletion	GCTCAGCCACCAGTTGCGCGCCGGCGTCGGCC
*cysG*-IF	SOEing PCR for *cysG* in-frame deletion	GGCCGACGCCGGCGCGCAACTGGTGGCTGAGC
*cysG*-OR	SOEing PCR for *cysG* in-frame deletion	GGATCCTGCGGCGCATCGAAGAC
*truB*-OF	SOEing PCR for *truB* in-frame deletion	GGATCCTGTTGATGTTGGCGG
*truB*-IR	SOEing PCR for *truB* in-frame deletion	GAGCGTGGCCCAGGTGTGATGCCGGAAGACAG
*truB*-IF	SOEing PCR for *truB* in-frame deletion	CTGTCTTCCGGCATCACACCTGGGCCACGCTC
*truB*-OR	SOEing PCR for *truB* in-frame deletion	AAGCTTACAGCCGTACCCAGC
PA-ArbM1	Arbitrary PCR for transposon insertion site mapping	CTTACCAGGCCACGCGTCGACTAGTACNNNNNNNNNNGATAT
PA-ArbM2	Arbitrary PCR for transposon insertion site mapping	CTTACCAGGCCACGCGTCGACTAGTAC
Arb1-BTK	Arbitrary PCR for transposon insertion site mapping	CACCGCTGCGTTCGGTCAAG
Arb2-BTK	Arbitrary PCR for transposon insertion site mapping	CGAACCGAACAGGCCTTATGTTCAATTC
Seq-BTK	Sequencing of arbitrary PCR amplicons	GGATGAAGTGGTTCGCATCCTC
SA-ArbA1	Arbitrary PCR for transposon insertion site mapping	GGCCACGCGTCGACTAGTCANNNNNNNNGATCA
SA-ArbA2	Arbitrary PCR for transposon insertion site mapping	GGCCACGCGTCGACTAGTCA
Arb1-Tn917	Arbitrary PCR for transposon insertion site mapping	CACCTGCAATAACCGTTACCTG
Arb2-Tn917	Arbitrary PCR for transposon insertion site mapping	TCACAATAGAGAGATGTCACCG
Seq-Tn917	Sequencing of arbitrary PCR amplicons	CCAATCACTCTCGGACAATAC

^a^Underlining denotes the engineered restriction enzyme sites
